# Supplementary material for: Perioperative Therapy for Non-Small Cell Lung Cancer with Immune Checkpoint Inhibitors
Source: Cancers (Basel). 2021 Aug 10;13(16):4035. doi: 10.3390/cancers13164035 (PMC8391213; doi:10.3390/cancers13164035)
Supplement: Supplementary file 1 [file cancers-13-04035-s001.zip › cancers-1321293-supplementary.pdf]

cancers

MDPI

Table S1 Clinical trials of neoadjuvant therapy using combination regimens of ICIs (phase 2/3)

Registration #	Trial	Therapy	Phase	N	Stage	Experimental arm	Control arm	Primary endpoint	Country
NCT02998528	Checkmate 816	ICI +chemo	3	358	IB to IIIA	nivolumab+PT-DC	PT-DC	EFS/pCR	Global
NCT03425643	KEYNOTE-671	ICI +chemo	3	786	II to IIIB(N2)	pembrolizumab+PT-DC +adjuvant pembrolizumab	placebo +PT-DC	EFS/OS	Global
NCT03456063	IMpower030	ICI +chemo	3	450	II to IIIB(T3N2)	atezolizumab+PT-DC	placebo +PT-DC	EFS	Global
NCT03800134	AEGEAN	ICI +chemo	3	800	II to III	durvalumab+PT-DC	placebo +PT-DC	MPR/EFS	Global
NCT04025879	Checkmate 77T	ICI +chemo	3	452	IIA(>4cm) to IIIB(T3N2)	nivolumab+PT-DC +adjuvant nivolumab	placebo +PT-DC	EFS	Global
NCT02716038	Columbia Univ.	ICI +chemo	2	30	IB to IIIA	atezolizumab +CBDCA/Nab-PTX(4times)	N/A	MPR	US
NCT02572843	SAKK16/14	ICI +chemo	2	68	IIIA(pN2)	durvalumab +CDDP/DTX (twice)	N/A	EFS	Switzerland
NCT03081689	NADIM	ICI +chemo	2	46	IIIA(pN2)	nivolumab +CBDCA/PTX (3times) +adjuvant nivolumab	N/A	PFS	Spain
NCT03366766	Thomas Jefferson Univ.	ICI +chemo	2	14	I(>=4cm) to IIIA	non-SQ: nivolumab +CDDP/PEM (3times) SQ: nivolumab +CDDP/GEM (3times)	N/A	MPR	US
NCT03480230	American Univ. of Beirut Medical Center	ICI +chemo	2	60	II or IIIA	avelumab+PT-DC (twice)	N/A	ORR	Jordan/ Lebanon
NCT03838159	NADIMII	ICI +chemo	2	90	IIIA, IIIB(T3N2)	nivolumab +CBDCA/PTX (3times) +adjuvant nivolumab	CBDCA/PTX +observation	pCR	Spain
NCT04326153	Jilin University	ICI +chemo	2	40	IIIA	sintilimab +CBDCA/albuminPTX (twice)	N/A	2-year DFS	China
NCT04061590	Univ. of California	ICI +chemo	2	84	I to IIIA	Arm A: pembrolizumab (twice) Arm B: pembrolizumab +CDDP/PEM (twice)	N/A	% of >=2-fold TIICs in post- vs. pre-treatment specimens	US
NCT03217071	Univ. of California	ICI +CRT	2	12	I to IIIA	pembrolizumab (twice) +/- SRT 12Gy	N/A	% of >=2-fold infiltrating CD3+ T cells from baseline	US
NCT03237377	Johns Hopkins Univ.	ICI +CRT	2	32	III	durvalumab+RT 45Gy durvalumab +tremelimumab +RT 45Gy	N/A	Toxities /Feasibility	US Canada
NCT03871153	HCRN LUN17-321	ICI +CRT	2	25	T1-4N2M0	durvalumab +CBDCA/PTX (3times) +RT 45-61.2Gy	N/A	pCR	US
NCT04202809	ESPADURVA	ICI +CRT	2	90	IIIA to IIIB	durvalumab +CDDP/DTX +RT 45Gy +adjuvant durvalumab	CRT	PFS	Germany
NCT04245514	SAKK16/18	ICI +CRT	2	90	T1-3&4(>7cm)N2M0	durvalumab +CDDP/DTXl (once) +RT: 20x2Gy(4w), 5x5Gy(1w), or 3x8Gy (1w)	N/A	EFS	Switzerland

NCT036 94236	Yonsei Univ.	ICI +CRT	1/2	39	III(N2)	durvalumab +CBDCA/PTX +RT 45Gy	N/A	pCR	Korea
JapicCTI-195069	WJOG12119L: SQUAT trial	ICI +CRT	1/2	31	IIIA(pN2)	durvalumab +CBDCA/PTX +RT 45Gy	N/A	MPR	Japan

ICI, immune checkpoint inhibitor; CRT, chemoradiotherapy; PT-DC, platinum-based doublet chemotherapy; CBDCA, carboplatin; PTX, paclitaxel; CDDP, cisplatin; DTX, docetaxel; PEM, pemetrexed; SRT, stereotactic radiotherapy; N/A, not applicable; EFS, event-free survival; OS, overall survival; MPR, major pathologic response; PFS, progression-free survival; ORR, overall response rate; pCR, pathological complete response; DFS, disease-free survival; TIIC, tumor-infiltrating immune cells
